# Embedding Age-Friendly Care using the 4Ms Framework in the Convenient Care Setting

**DOI:** 10.1093/geroni/igaa057.3320

**Published:** 2020-12-16

**Authors:** Sherry Greenberg, Lilia Pino, Mary McCormack, Evelyn Duffy, Elizabeth Zimmermann, Robin Hughes, Anne Pohnert, Mary Dolansky

**Affiliations:** 1 Seton Hall University College of Nursing, South Orange, New Jersey, United States; 2 CVS Health Minute Clinic, Woonsocket, Rhode Island, United States; 3 Case Western Reserve University, Cleveland, Ohio, United States

## Abstract

This late-breaker presentation highlights the implementation science plan for Age-Friendly care in CVS Health MinuteClinic’s convenient care clinics. This project, a partnership between MinuteClinic, Case Western Reserve University Frances Payne Bolton School of Nursing, and the Institute for Healthcare Improvement, funded by The John A. Hartford Foundation, increases the number of care providers trained in the provision of Age-Friendly care using the 4Ms Framework: What Matters, Medication, Mentation, and Mobility. The implementation team learned the MinuteClinic usual processes, then developed strategies for successful Age-Friendly care implementation. Data from 21 nurse practitioner, 5 patient interviews, educator focus groups and 14 site visits revealed time, resources, and perceived value as barriers to implementation. Most reported increased knowledge and willingness to change practice. Based on the Plan-Do-Study-Act change process data, the implementation team developed solutions that addressed gaps leading to the development of practice-based tools for successful Age-Friendly care project adoption and implementation.

